# Real‑world safety evaluation of tranexamic acid: Signal detection from FAERS and VigiAccess databases

**DOI:** 10.1371/journal.pone.0353459

**Published:** 2026-07-10

**Authors:** Jing Feng, Chiwei Guo, Shujuan Zhao

**Affiliations:** 1 Department of Pharmacy, Henan Provincial People’s Hospital, People’s Hospital of Zhengzhou University, School of Clinical Medicine, Henan University, Zhengzhou, Henan, China; 2 Department of Endocrinology, The First People’s Hospital of Nankang District, Ganzhou, Jiangxi, China; Deccan School of Pharmacy, INDIA

## Abstract

**Background:**

Tranexamic acid (TXA) is an antifibrinolytic agent commonly used to mitigate blood loss across various medical indications. Despite its widespread use, comprehensive data on its safety profile remain limited. This study aimed to systematically evaluate adverse events (AEs) associated with TXA.

**Methods:**

Adverse event reports were extracted from the U.S. Food and Drug Administration’s Adverse Event Reporting System (FAERS) and the VigiAccess databases. Disproportionality analyses were conducted using reporting odds ratio (ROR), proportional reporting ratio (PRR), the Medicines and Healthcare products Regulatory Agency (MHRA) method, Bayesian confidence propagation neural network (BCPNN), and multi-item gamma Poisson shrinker (MGPS).

**Results:**

A total of 17,787 TXA-related AE reports were identified. A higher proportion of reports involved females, with older adults (≥ 65 years) accounting for the largest proportion in FAERS and younger individuals (18–44 years) in VigiAccess. Overlapping PTs, including seizures, pulmonary embolism and anaphylactic reactions, were identified. Significant differences for TXA-related AEs were found by gender, age and death outcomes. Most AEs occurred within the first month, with an early failure pattern.

**Conclusion:**

This study provides evidence to weigh risks and benefits of TXA by comprehensive assessment of safety profile for TXA. These findings provide valuable references for future pharmacovigilance research on TXA.

## Introduction

Tranexamic acid (TXA), an antifibrinolytic agent that inhibits plasminogen activation, has been widely used in clinical practice for decades [1]. Since its introduction in the 1960s, TXA has demonstrated efficacy in reducing blood loss across a variety of medical scenarios, including trauma, surgery, obstetrics, gynecology, and other bleeding disorders, making it an essential tool in the global management of hemorrhage [2,3]. Moreover, with its inclusion in the World Health Organization (WHO)’s essential medicines list, TXA has become a cornerstone for managing hemorrhagic conditions globally [4].

However, current evidence on TXA’s safety primarily comes from clinical trials [4–6], which are often constrained by small sample sizes, short follow-up periods, or narrowly defined patients. Real-world pharmacovigilance studies provide a complementary perspective to identify adverse events (AEs) signals related with TXA in broader populations, including those not studied in pre-marketing trials [7]. While numerous studies have confirmed TXA’s hemostatic benefits [8,9], its safety profile, including AE signal distribution, subgroup-specific risks, and time-to-onset (TTO) patterns, has yet to be characterized, particularly across multiple pharmacovigilance databases.

In this study, we aim to assess TXA’s safety profile using the U.S. Food and Drug Administration’s Adverse Event Reporting System (FAERS) and VigiAccess database. By using disproportionality algorithms and stratified analyses, we sought to identify significant AE signals at both the system organ class (SOC) and preferred term (PT) levels, determine subgroup-specific risks stratified by age, gender, and fatal outcomes, and perform TTO analysis of TXA-related AEs. This multidimensional approach provides evidence for clinical decision-making and helps to optimize the management of hemorrhagic disorders.

## Materials and methods

### Data source

Data were collected from two pharmacovigilance databases: the FAERS database, which covers reports from the first quarter of 2004 to the third quarter of 2024, and the VigiAccess database, which includes data from the drug’s market introduction to December 29, 2024. In FAERS, spontaneous AE reports were submitted by healthcare professionals, patients, and drug manufacturers [10], while VigiAccess is a user-friendly portal, allowing to search drug safety reports received by the Uppsala Monitoring Centre from around the world [11]. Although individual case safety reports (ICSRs) are not publicly accessible via VigiAccess, aggregated drug–event frequency data were retrieved from the portal. Approximate contingency tables were reconstructed from these aggregate counts to support exploratory signal detection.

The search was conducted using generic nomenclature, with AEs systematically categorized and encoded according to the Medical Dictionary for Regulatory Activities (MedDRA, version 27.1) [12]. These coded terms were arranged in a hierarchy of categories to facilitate standardized classification of specific categories (e.g., narrow PT and broad SOC).

Since this study was based on anonymous data available to the public, institutional review board approval and informed patient consent were not necessary.

### Study design

This study employed a case/non-case approach, similar to a case-control design, to assess the safety profile of TXA [13]. For FAERS, a total of 21,964,449 reports were initially retrieved. Deduplication was performed following FDA guidance using three fields from the DEMO table: PRIMARYID, CASEID, and FDA_DT. For reports sharing the same CASEID, the record with the most recent FDA_DT was retained; where both CASEID and FDA_DT were identical, the record with the highest PRIMARYID was kept. After deduplication, 3,686,206 duplicate records were excluded, yielding 18,278,243 unique reports for background analysis, of which 1,780 were TXA-related AE reports included in this study.

For VigiAccess, as only aggregate-level frequency counts are available rather than individual ICSRs, approximate 2 × 2 contingency tables were constructed from the reported drug–event frequencies to enable exploratory disproportionality analysis. These results are considered complementary to FAERS-based signals. After deduplication and data screening, a total of 17,787 TXA-related AE reports were identified from the two databases for further analysis.

Within this cohort, descriptive analysis and disproportionality methods were utilized to identify TXA-related AE signals. If the proportion of AEs is higher in patients exposed to TXA (cases) compared to those who were not exposed (non-cases), a correlation between the drug and the event can be assumed, suggesting a disproportionality signal. It should be noted that this design does not adjust for underlying indication or disease severity. As TXA is predominantly used in high-acuity settings, observed signals may partly reflect the background risk of the treated population rather than drug-specific effects, and should be interpreted as hypothesis-generating.

### Descriptive analyses

Descriptive analyses were performed to summarize the demographic characteristics of TXA-related AE reports. Variables included gender, age, reporting year, route of administration, reported countries, indications, outcomes, weight, etc. Categorical variables were presented as frequencies and percentages, while continuous variables with non-normal distributions were reported as medians with interquartile ranges (IQR).

### Disproportionality analysis

Formal disproportionality analysis was conducted on FAERS data using a combination of five algorithms, based on individual-level ICSRs and full contingency tables. For VigiAccess, exploratory signal detection was performed using approximate contingency tables reconstructed from aggregated frequency counts, with the same algorithm parameter thresholds as FAERS. The five algorithms applied were: reporting odds ratio (ROR), proportional reporting ratio (PRR), Medicines and Healthcare Products Regulatory Agency (MHRA) criteria, Bayesian confidence propagation neural network (BCPNN), and the multi-item gamma Poisson shrinker (MGPS) algorithm (S1 Table and S2 Table) [14–18]. A potential positive signal was identified if TXA demonstrated a positive association across all five algorithms: (1) For ROR, the frequency of AE occurrences (a) ≥ 3, with the lower bound of the 95% confidence interval (CI) for ROR > 1; (2) For PRR, a ≥ 3, with the lower bound of the 95% CI for PRR > 1; (3) For MHRA, a ≥ 3, PRR ≥ 2, and chi-square (χ^2^) ≥ 4; (4) For BCPNN, the lower bound of the 95% CI for the information component (IC025) > 0; (5) For MGPS, the lower bound of the 95% CI for the empirical Bayesian geometric mean (EBGM05) > 2. This conservative approach requires simultaneous positivity across all five algorithms, combined with important medical event (IME) classification, to maximize specificity and minimize false-positive signals. Although this strategy may reduce sensitivity, concordance across distinct algorithms strengthens the evidence for retained signals. PTs meeting signal thresholds with 3–9 reports are acknowledged as potential signals but were not included in the primary tables due to statistical imprecision. These represent areas for future investigation.

To further refine the analysis, an IME list was introduced at the PT level. The IME list, published by the European Union and updated biannually in alignment with MedDRA (version 27.1), was used to refine PT-level signals; only PTs meeting predefined thresholds and classified as IMEs were included in the final analysis. To ensure statistical stability, PTs with fewer than 10 total reports were excluded from the final tabulated results.

### Difference analysis

In FAERS, difference analysis was conducted to explore potential variations in AE signals across demographic and clinical parameters. Specifically, analyses were performed by gender (male vs. female), age (< 65 years vs. ≥ 65 years), and fatal outcomes (death vs. non-death). To visualize these differences, “volcano plots” were constructed, providing a graphical representation of AE signal variations. In the volcano plots, the -log10 of the *P*-value was plotted on the y-axis, while the log2 of the ROR was displayed on the x-axis. Between-subgroup differences in ROR were assessed for each PT using chi-square tests, with Yates’ continuity correction or Fisher’s exact test applied as appropriate. To account for multiple comparisons, Benjamini–Hochberg false discovery rate (FDR) correction was applied across all PTs within each subgroup analysis. The volcano plots display unadjusted P-values for visual clarity; FDR-corrected P-values were used to determine statistical significance, with FDR-adjusted *P* < 0.05 considered significant.

### TTO analysis

To examine variations in AE occurrences over time, we performed TTO analysis. TTO was defined as the interval between TXA initiation and AE occurrence, calculated from drug start date and event date fields. Records with missing, or erroneous dates were excluded. Cases with a TTO of 0 days were retained, as same-day events are clinically plausible for intravenous administration (e.g., infusion-related reactions). However, we acknowledge that 0-day records may also reflect date imprecision or reporting conventions rather than true same-day onset. Of the 1,780 FAERS reports, 391 (21.97%) contained evaluable TTO data and were included in this analysis; the remaining 1,389 (78.03%) were excluded due to missing date information. Cumulative distribution curves were plotted to visualize TTO patterns, and gender-stratified analyses were performed to explore potential differences.

In addition to median onset times and IQR, Weibull’s shape parameter (WSP) test was applied to classify TTO patterns [19,20]. The WSP includes parameters of scale (α) and shape (β). The shape parameter β was performed to classify AE risk patterns: (1) Early failure, characterized by a decreased AE hazard over time (β < 1, and 95% confidence interval [CI] < 1); (2) Random failure, characterized by a constant AE hazard over time (β is equal to or close to 1, and 95% CI contains 1); (3) Wear-out failure, characterized by an increased AE hazard over time (β > 1, and 95% CI > 1) [21].

Furthermore, in TTO analysis, we introduced Standardized MedDRA Queries (SMQs). The SMQs consist of groupings of MedDRA terms ordinarily at the PT level that relate to a defined medical condition or area of interest [22].

### Statistics analysis and reporting guidelines

All statistical analyses and visualizations were carried out using SAS software (version 9.4; SAS Institute Inc., Cary, NC), R software (version 4.4.2; R Foundation for Statistical Computing, Vienna, Austria), Python (version 3.10; Python Software Foundation, Wilmington, United States), and Prism (version 9.5; GraphPad Software, San Diego, CA). A *P*-value < 0.05 was considered statistically significant.

This study followed the REporting of A Disproportionality analysis for drUg Safety signal detection using individual case safety reports in PharmacoVigilance (READUS-PV) guidelines (S3 Table) [23].

## Results

### Descriptive analysis

A total of 17,787 TXA-related AE reports were collected from the two pharmacovigilance databases: 1,780 reports from FAERS and 16,007 reports from VigiAccess (Table 1). The VigiAccess database provided demographic characteristics based on four dimensions: gender, age, reporting year, and continent. Excluding reports with unknown gender, the female-to-male ratio for TXA-related AEs was approximately 1.80–2.09 times higher in both databases (53.26% vs. 29.66% in FAERS; 65.96% vs. 31.63% in VigiAccess). Regarding age, FAERS reported a median age of 52.5 years (IQR: 37.0–70.0), with patients aged 65 and older accounting for the highest reporting proportion of TXA-related AEs (25.51%, n = 454). Conversely, in VigiAccess, the 18–44 age group reported the highest proportion of TXA-related AEs, representing 36.62% (n = 5,862). In terms of geographical distribution, the majority of reports came from the Americas (45.11%) in FAERS and Asia (72.14%) in VigiAccess. TXA-related AE reports exhibited an increasing trend in recent years (S1 Fig and S2 Fig), with more than half of the reports from both databases occurring between 2019 and 2024 (FAERS: 60.11%; VigiAccess: 57.46%).

**Table 1:** Demographic characteristics of reports with TXA.

Characteristics	FAERS(n = 1780)	VigiAccess(n = 16,007)	Characteristics	FAERS(n = 1780)
Gender (n, %)			Country of the reporters (n, %)	
Female	948 (53.26)	10,558 (65.96)	United States	701 (39.38)
Male	528 (29.66)	5,063 (31.63)	United Kiongdom	340 (19.10)
Unknown	304 (17.08)	386 (2.41)	Germany	111 (6.24)
Age			Canada	90 (5.06)
N (Missing) (n, %)	1,342 (438)	/	Netherlands	61 (3.43)
Median (IQR) (y)	52.5 (37.0,70.0)	/	Indications	
Min, Max (y)	0.00, 98.00	/	Not Specified	324 (18.20)
< 18 (n, %)	114 (6.40)	695 (4.34)	Product used for unknown indication	213 (11.97)
18-44 (n, %)	365 (20.51)	5,862 (36.62)	Heavy menstrual bleeding	204 (11.46)
45-64 (n, %)	409 (22.98)	5,409 (33.79)	Haemorrhage	136 (7.64)
≥ 65 (n, %)	454 (25.51)	2,790 (18.55)	Haemorrhage prophylaxis	94 (5.28)
Not specified (n, %)	438 (24.61)	1,071 (6.69)	Serious reports (n, %)	
Reporter year (n, %)			Serious	1,678 (94.27)
2004-2008	59 (3.31)	181 (1.13)	Non-Serious	102 (5.73)
2009-2013	247 (13.88)	839 (5.24)	Outcome (n, %)	
2014-2018	404 (22.70)	5,270 (32.92)	Life-threatening	272 (15.28)
2019-2024	1,070 (60.11)	9,198 (57.46)	Hospitalization – initial or prolonged	570 (32.02)
Reporter (n, %)			Disability	64 (3.60)
Consumer	172 (9.66)	/	Death	164 (9.21)
Lawyer	3 (0.17)	/	Congenital anomaly	5 (0.28)
Not specified	91 (5.11)	/	Required intervention to prevent permanent impairment/damage	13 (0.73)
Other health-professional	297 (16.69)	/	Other	1,200 (67.42)
Pharmacist	570 (32.02)	/	Occurrence time	
Physician	647 (36.35)	/	Median (IQR) (days)	0.0 (0.0, 5.0)
Continent (n, %)		/	0-30 d (n, %)	350 (19.66)
Africa	33 (1.85)	224 (1.4)	31-60 d (n, %)	11 (0.62)
Americas	803 (45.11)	1,325 (8.28)	61-90 d (n, %)	5 (0.28)
Asia	159 (8.93)	11,547 (72.14)	91-120 d (n, %)	6 (0.34)
Europe	711 (39.94)	2,677 (16.72)	121-150 d (n, %)	3 (0.17)
Oceania	61 (3.43)	234 (1.46)	151-180 d (n, %)	3 (0.17)
Route (n, %)			181-360 d (n, %)	6 (0.34)
Not specified	885 (49.72)	/	> 360 d (n, %)	7 (0.39)
Intravenous	372 (20.90)	/	Not specified (n, %)	1,389 (78.03)
Oral	284 (15.96)	/	Weight	
Intrathecal	98 (5.51)	/	N (Missing)	482 (1,298)
Intravenous bolus	55 (3.09)	/	Median (IQR) (kg)	72.90 (59.87, 87.50)

TXA, Tranexamic acid; FAERS, The U.S. Food and Drug Administration (FDA)’s Adverse Event Reporting System; N, Number of cases; IQR, Interquartile range.

Further, in FAERS, over four-fifths of all reports (85.06%) were submitted by healthcare professionals, with the majority of reports originating from the United States (39.38%). The most common route of administration was not specified (49.72%), followed by intravenous (20.90%). Notably, 94.27% of the reports were classified as serious, with hospitalization being the most frequently reported outcome (32.02%). In terms of indications, excluding not specified or unknown indications, the most common TXA-related indication was heavy menstrual bleeding (n = 204), followed by hemorrhage (n = 136) and hemorrhage prophylaxis (n = 94).

### Distribution at SOC level for TXA

**Fig 1:**
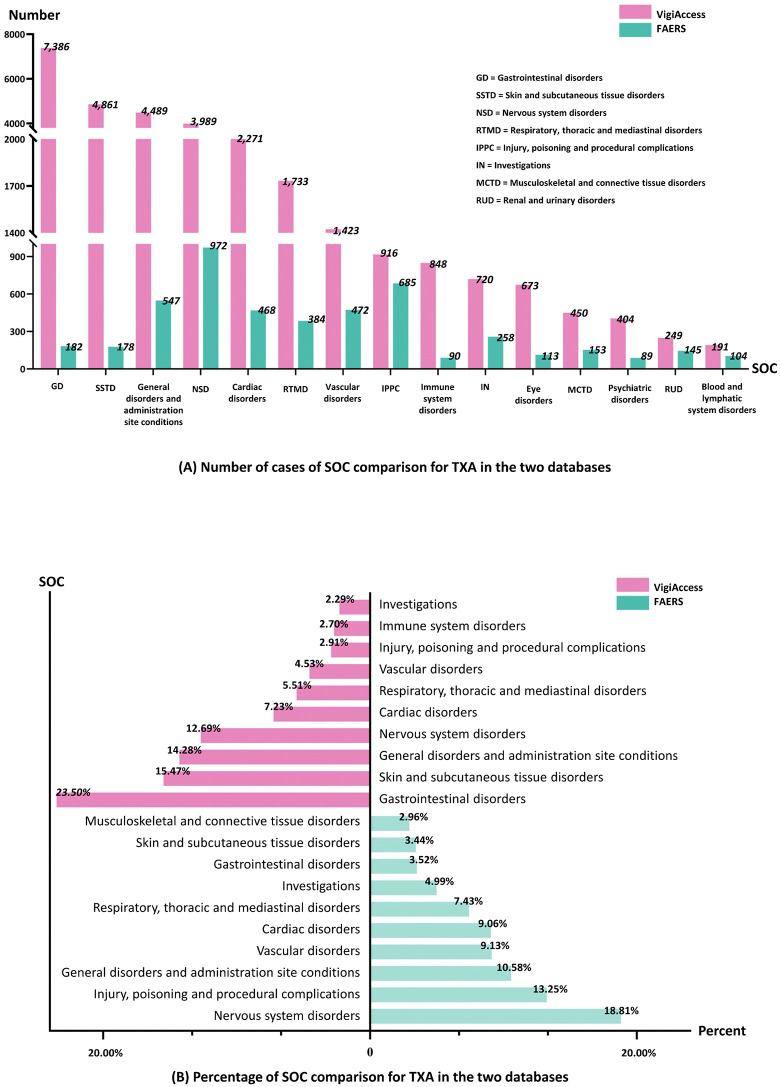
Distribution of signals at the SOC level for TXA in the two databases. TXA, Tranexamic acid; FAERS, The U.S. Food and Drug Administration (FDA)’s Adverse Event Reporting System; SOC, System organ class.

AEs were categorized based on the SOC classification. In VigiAccess, the most frequently reported AEs were related to gastrointestinal disorders (n = 7,386, 23.50%), skin and subcutaneous tissue disorders (n = 4,861, 15.47%), and general disorders and administration site conditions (n = 4,489, 14.28%). In contrast, in FAERS, the top three SOC categories for TXA-related AEs were nervous system disorders (n = 972, 18.81%), injury, poisoning, and procedural complications (n = 685, 13.25%), and general disorders and administration site conditions (n = 547, 10.58%). Despite differences in ranking, a considerable overlap was observed in the SOC levels between the two databases (Fig 1).

### Distribution at PT level for TXA

We analyzed positive signals at the PT level, focusing on PTs with at least 10 reports. The cumulative number of reports for TXA-related PTs was 1,109 in FAERS and 2,807 in VigiAccess. A total of 34 PT signals were identified in FAERS, while 25 positive PT signals were observed in VigiAccess. The analysis highlighted the most frequently reported PTs and those with the highest signal intensity.

**Fig 2:**
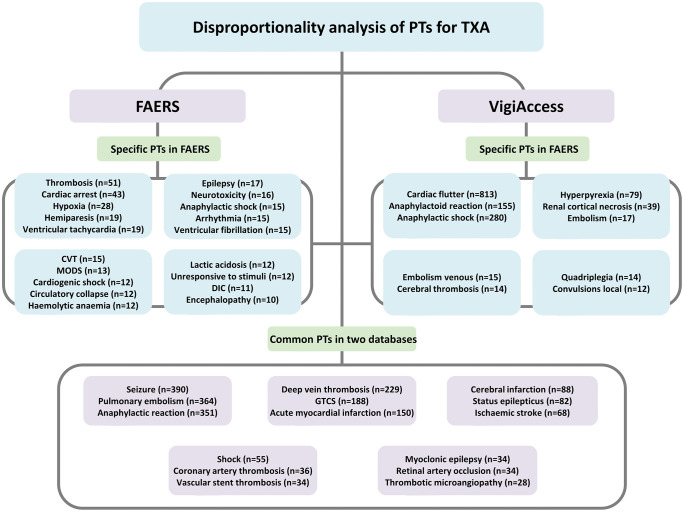
Distribution of signals at the PT level for TXA in the two databases. PT, Preferred term; TXA, Tranexamic acid; FAERS, The U.S. Food and Drug Administration (FDA)’s Adverse Event Reporting System; CVT, Cardiac ventricular thrombosis; MODS, Multiple organ dysfunction syndrome; DIC, Disseminated intravascular coagulation; GTCS, Generalised tonic-clonic seizure.

In FAERS (S3 Fig), the three most frequently reported PTs for TXA were seizure (n = 151), pulmonary embolism (PE) (n = 119), and generalized tonic-clonic seizure (GTCS) (n = 89). The top three PT signals with the highest ROR were myoclonic epilepsy (ROR = 165.01), coronary artery thrombosis (ROR = 123.15), vascular stent thrombosis (ROR = 118.45) (Table 2). In VigiAccess (S4 Fig), the three most frequently reported PT signals for TXA were cardiac flutter (n = 813), anaphylactic reaction (n = 299), anaphylactic shock (n = 280). The PT signals with the highest ROR in VigiAccess were renal cortical necrosis (ROR = 542.55), myoclonic epilepsy (ROR = 37.36), cardiac flutter (ROR = 35.99) (Table 3). Of the 25 PT signals identified in VigiAccess, 15 (60%) were also detected in FAERS. When ranked by case numbers, the top three overlapping PTs were seizure (n = 390), PE (n = 364), and anaphylactic reaction (n = 351). In addition to the overlapping PTs, 19 unique PTs were reported in FAERS, while 10 unique PTs were identified in VigiAccess (Fig 2).

**Table 2:** Identification of PT signals for TXA using disproportionality analysis in FAERS.

PTs and in accordance with the IME standards	Reports	ROR (95% CI lower)	PRR (95% CI lower)	MHRA (χ^2^)	BCPNN (IC025)	MGPS (EBGM05)
Seizure	151	10.62 (9.03)	10.34 (8.83)	1276.04	3.37 (3.04)	10.33 (8.78)
Pulmonary embolism	119	14.75 (12.30)	14.43 (12.08)	1487.90	3.85 (3.43)	14.41 (12.02)
Generalised tonic-clonic seizure	89	41.45 (33.60)	40.75 (33.15)	3439.09	5.34 (4.51)	40.60 (32.91)
Acute myocardial infarction	84	32.82 (26.45)	32.31 (26.12)	2541.83	5.01 (4.24)	32.21 (25.95)
Deep vein thrombosis	55	9.69 (7.43)	9.60 (7.38)	423.57	3.26 (2.67)	9.59 (7.35)
Anaphylactic reaction	52	11.99 (9.12)	11.88 (9.06)	517.74	3.57 (2.90)	11.86 (9.03)
Thrombosis	51	7.48 (5.68)	7.42 (5.65)	283.45	2.89 (2.32)	7.42 (5.63)
Status epilepticus	47	51.09 (38.31)	50.63 (38.075)	2276.07	5.66 (4.21)	50.39 (37.79)
Cardiac arrest	43	6.09 (4.51)	6.05 (4.49)	181.38	2.60 (2.00)	6.05 (4.48)
Cerebral infarction	35	16.79 (12.04)	16.68 (11.99)	515.39	4.06 (3.05)	16.66 (11.94)
Hypoxia	28	9.75 (6.72)	9.70 (6.70)	218.45	3.28 (2.36)	9.69 (6.69)
Ischaemic stroke	26	17.17 (11.68)	17.09 (11.64)	393.29	4.09 (2.86)	17.06 (11.60)
Coronary artery thrombosis	22	123.15 (80.82)	122.63 (80.62)	2623.62	6.92 (3.68)	121.23 (79.56)
Vascular stent thrombosis	20	118.45 (76.16)	117.99 (76.00)	2294.34	6.87 (3.53)	116.69 (75.04)
Hemiparesis	19	12.89 (8.21)	12.85 (8.20)	207.38	3.68 (2.36)	12.83 (8.18)
Ventricular tachycardia	19	13.58 (8.65)	13.53 (8.63)	220.26	3.76 (2.41)	13.51 (8.61)
Myoclonic epilepsy	19	165.01 (104.79)	164.40 (104.584)	3038.32	7.34 (3.51)	161.89 (102.81)
Retinal artery occlusion	18	89.15 (56.01)	88.84 (55.91)	1550.33	6.46 (3.31)	88.11 (55.36)
Epilepsy	17	6.88 (4.27)	6.86 (4.27)	85.135	2.78 (1.69)	6.86 (4.26)
Neurotoxicity	16	11.73 (7.18)	11.70 (7.17)	156.41	3.55 (2.14)	11.69 (7.15)
Anaphylactic shock	15	7.24 (4.36)	7.22 (4.35)	80.32	2.85 (1.65)	7.21 (4.34)
Arrhythmia	15	3.66 (2.21)	3.66 (2.21)	28.96	1.87 (0.93)	3.66 (2.20)
Ventricular fibrillation	15	15.80 (9.52)	15.76 (9.50)	207.06	3.98 (2.31)	15.74 (9.48)
Cardiac ventricular thrombosis	15	40.38 (24.30)	40.27 (24.27)	572.27	5.33 (2.82)	40.12 (24.15)
Thrombotic microangiopathy	14	18.41 (10.89)	18.37 (10.88)	229.52	4.20 (2.34)	18.34 (10.85)
Multiple organ dysfunction syndrome	13	3.46 (2.01)	3.45 (2.01)	22.66	1.79 (0.78)	3.45 (2.00)
Cardiogenic shock	12	10.54 (5.98)	10.52 (5.98)	103.30	3.39 (1.80)	10.51 (5.96)
Circulatory collapse	12	8.07 (4.58)	8.06 (4.58)	74.14	3.01 (1.58)	8.05 (4.57)
Haemolytic anaemia	12	15.81 (8.97)	15.78 (8.96)	165.84	3.98 (2.08)	15.75 (8.94)
Lactic acidosis	12	4.68 (2.66)	4.68 (2.66)	34.67	2.22 (1.06)	4.67 (2.65)
Unresponsive to stimuli	12	5.52 (3.13)	5.51 (3.13)	44.26	2.46 (1.23)	5.50 (3.12)
Disseminated intravascular coagulation	11	9.01 (4.99)	8.99 (4.98)	78.11	3.17 (1.60)	8.99 (4.97)
Shock	11	5.88 (3.26)	5.87 (3.25)	44.48	2.55 (1.23)	5.87 (3.25)
Encephalopathy	10	4.91 (2.64)	4.90 (2.64)	31.02	2.29 (0.98)	4.90 (2.63)

Note: If the number of PTs was less than 10, it was excluded from the analysis.

PT, Preferred term; TXA, Tranexamic acid; FAERS, The U.S. Food and Drug Administration (FDA)’s Adverse Event Reporting System; IME, Important medical event; ROR, Reporting odds ratio; CI, Confidence interval; PRR, Proportional reporting ratio; MHRA, Medicines and healthcare products regulatory agency; BCPNN, Bayesian confidence propagation neural network; IC, Information component; MGPS, Multi-item gamma poisson shrinker; EBGM, Empirical bayesian geometric mean.

**Table 3:** Identification of PT signals for TXA using disproportionality analysis in VigiAccess.

PTs and in accordance with the IME standards^#^	Reports	ROR (95% CI lower)	PRR (95% CI lower)	MHRA (χ^2^)	BCPNN (IC025)	MGPS (EBGM05)
Cardiac flutter	813	35.99 (33.56)	35.09 (32.78)	26694.72	5.12 (4.96)	34.77 (32.42)
Anaphylactic reaction	299	5.14 (4.59)	5.10 (4.56)	986.74	2.35 (2.16)	5.10 (4.55)
Anaphylactic shock	280	6.57 (5.84)	6.52 (5.80)	1307.23	2.70 (2.50)	6.51 (5.78)
Pulmonary embolism	245	6.27 (5.53)	6.23 (5.50)	1074.87	2.64 (2.42)	6.22 (5.48)
Seizure	239	2.73 (2.41)	2.72 (2.40)	260.43	1.44 (1.25)	2.72 (2.39)
Deep vein thrombosis	174	6.11 (5.26)	6.08 (5.24)	737.69	2.60 (2.34)	6.07 (5.23)
Anaphylactoid reaction	155	7.20 (6.14)	7.17 (6.12)	821.34	2.84 (2.55)	7.15 (6.11)
Generalised tonic-clonic seizure	99	7.36 (6.04)	7.34 (6.03)	541.27	2.87 (2.50)	7.33 (6.01)
Hyperpyrexia	79	4.16 (3.34)	4.15 (3.33)	189.14	2.05 (1.67)	4.15 (3.33)
Acute myocardial infarction	66	7.27 (5.71)	7.25 (5.70)	355.17	2.86 (2.37)	7.24 (5.69)
Cerebral infarction	53	6.08 (4.64)	6.07 (4.64)	224.35	2.60 (2.08)	6.07 (4.63)
Shock	44	4.96 (3.69)	4.96 (3.69)	138.93	2.31 (1.76)	4.95 (3.69)
Ischaemic stroke	42	7.08 (5.23)	7.07 (5.23)	218.68	2.82 (2.19)	7.06 (5.22)
Renal cortical necrosis	39	542.55 (387.74)	541.87 (387.40)	18397.77	8.89 (4.72)	473.61 (338.47)
Status epilepticus	35	9.73 (6.98)	9.72 (6.98)	273.21	3.28 (2.48)	9.70 (6.96)
Embolism	17	4.91 (3.05)	4.91 (3.05)	52.82	2.29 (1.33)	4.90 (3.05)
Retinal artery occlusion	16	19.72 (12.06)	19.71 (12.06)	282.62	4.29 (2.52)	19.61 (12.00)
Embolism venous	15	15.94 (9.60)	15.94 (9.60)	209.11	3.99 (2.32)	15.87 (9.56)
Myoclonic epilepsy	15	37.36 (22.46)	37.34 (22.46)	525.28	5.21 (2.78)	36.98 (22.24)
Cerebral thrombosis	14	10.79 (6.38)	10.78 (6.38)	123.90	3.43 (1.96)	10.75 (6.36)
Coronary artery thrombosis	14	17.39 (10.29)	17.39 (10.28)	215.20	4.11 (2.30)	17.31 (10.24)
Quadriplegia	14	11.39 (6.74)	11.39 (6.74)	132.30	3.51 (2.00)	11.36 (6.72)
Thrombotic microangiopathy	14	5.73 (3.39)	5.73 (3.39)	54.549	2.52 (1.38)	5.72 (3.39)
Vascular stent thrombosis	14	29.67 (17.53)	29.66 (17.53)	384.62	4.88 (2.60)	29.43 (17.39)
Convulsions local	12	12.83 (7.28)	12.83 (7.28)	130.44	3.68 (1.94)	12.79 (7.25)

Note: If the number of PTs was less than 10, it was excluded from the analysis. Results are based on approximate contingency tables reconstructed from aggregated VigiAccess frequency data. These results should be interpreted as exploratory findings.

PT, Preferred term; TXA, Tranexamic acid; IME, Important medical event; ROR, Reporting odds ratio; CI, Confidence interval; PRR, Proportional reporting ratio; MHRA, Medicines and healthcare products regulatory agency; BCPNN, Bayesian confidence propagation neural network; IC, Information component; MGPS, Multi-item gamma poisson shrinker; EBGM, Empirical bayesian geometric mean.

### Difference analysis

**Fig 3:**
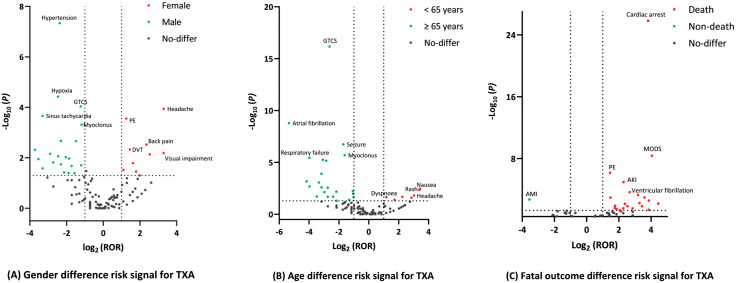
Difference analysis based on gender, age and fatal outcomes in FAERS. Note: Statistical significance was assessed using chi-square tests (with Yates’ continuity correction or Fisher’s exact test for sparse data), with Benjamini–Hochberg false discovery rate (FDR) correction applied for multiple comparisons (FDR-adjusted *P* < 0.05). FAERS, The U.S. Food and Drug Administration (FDA)’s Adverse Event Reporting System; TXA, Tranexamic acid; ROR, Reporting odds ratio; GTCS, generalised tonic-clonic seizure; PE, Pulmonary embolism; DVT, Deep vein thrombosis; MODS, Multiple organ dysfunction syndrome; AKI, Acute kidney injury; AMI, Acute myocardial infarction.

In exploratory subgroup analyses, females showed higher reporting disproportionality for headache, PE, and back pain, whereas males demonstrated higher signals for hypertension, hypoxia, and GTCS (Fig 3A). Age-based analysis suggested that individuals aged ≥ 65 years may have higher disproportionality signals for AEs such as GTCS, atrial fibrillation, and seizure, while younger individuals (< 65 years) showed higher reporting proportions for nausea, rash, and headache (Fig 3B). These subgroup findings are hypothesis-generating and should be interpreted with caution given the exploratory nature of the analyses. In the analysis of fatal outcomes, certain AEs were more frequently reported in association with death. Cardiac arrest, multiple organ dysfunction syndrome (MODS), and PE showed higher reporting disproportionality in the death group, while acute myocardial infarction (AMI) was more frequently reported in the non-death group (Fig 3C). These findings should be interpreted with caution, as patients who died may differ substantially from survivors in terms of disease severity, clinical indication, comorbidities, and concomitant medications.

### TTO analysis

**Fig 4:**
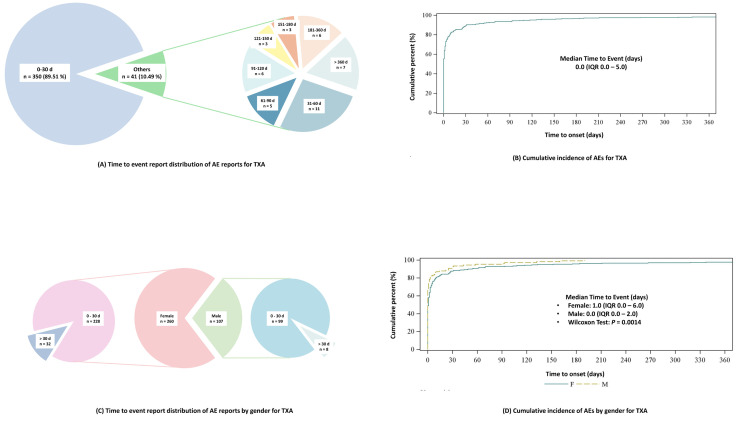
Time-to-onset analysis and cumulative incidence of AEs for TXA. AE, Adverse event; TXA, Tranexamic acid; IQR, Interquartile range.

In FAERS, after removing duplicates and erroneous reports, 391 cases provided TTO data. The majority of AEs occurred within the first month of treatment (n = 350, 89.51%) (Fig 4A), with a median time to event of 0 days (IQR 0.0–5.0 days) (Fig 4B). However, TTO data were available for only 391 of 1,780 cases (21.97%) and a substantial proportion had a recorded onset of 0 days, which may partly reflect same-day reporting conventions or date imprecision rather than true immediate onset. Among cases with evaluable TTO, Weibull shape parameter values (β < 1) were consistent with an early failure pattern across all SMQ categories (Table 4).

**Table 4:** TTO analysis of the overall AEs related with TXA and SMQs related to TXA in FAERS.

Item	Effective records of TTO time (n)	TTO	Weibull distribution				Failure type
		(days)	Scale parameter		Shape parameter		
		Median (IQR)	α	95% CI	β	95% CI	
**Overall AEs induced by TXA**	391	0.0 (0.0, 5.0)	25.07	17.71–35.47	0.45	0.41–0.50	Early failure
**Allergy**							
Hypersensitivity (SMQ)	86	0.0 (0.0, 1.0)	19.60	8.52–45.11	0.52	0.38–0.71	Early failure
Anaphylactic reaction (SMQ)	31	0.0 (0.0, 0.0)	–	–	–	–	–
Anaphylactic/anaphylactoid shock conditions (SMQ)	29	0.0 (0.0, 0.0)	–	–	–	–	–
**Thromboemboism**							
Embolic and thrombotic events, vessel type unspecified and mixed arterial and venous (SMQ)	55	2.0 (0.0, 9.0)	24.34	9.87–59.98	0.39	0.31–0.49	Early failure
Embolic and thrombotic events, venous (SMQ)	51	4.0 (1.0, 14.0)	20.03	10.49–38.26	0.49	0.40–0.59	Early failure
Embolic and thrombotic events, arterial (SMQ)	21	3.0 (0.0, 28.0)	52.17	14.61–186.32	0.44	0.30–0.65	Early failure
**Neuromuscular**							
Convulsions (SMQ)	53	0.0 (0.0, 0.0)	10.44	3.00–36.38	0.50	0.33–0.77	Early failure
Generalised convulsive seizures following immunisation (SMQ)	51	0.0 (0.0, 0.0)	10.44	3.00–36.38	0.50	0.33–0.77	Early failure
**Vascular**							
Ischaemic central nervous system vascular conditions (SMQ)	41	3.0 (1.0, 9.0)	17.28	8.43–35.42	0.52	0.41–0.67	Early failure
Haemorrhagic central nervous system vascular conditions (SMQ)	20	2.5 (0.0, 4.0)	10.51	4.07–27.16	0.61	0.42–0.89	Early failure
Angioedema (SMQ)	22	0.5 (0.0, 4.0)	9.79	3.50–27.39	0.61	0.39–0.95	Early failure
**Others**							
Gastrointestinal nonspecific symptoms and therapeutic procedures (SMQ)	38	1.0 (0.0, 7.0)	28.86	10.99–75.77	0.48	0.36–0.66	Early failure
Haemorrhage terms (excl laboratory terms) (SMQ)	27	0.0 (0.0, 8.0)	38.40	9.40–156.80	0.47	0.31–0.72	Early failure
Shock-associated circulatory or cardiac conditions (excl torsade de pointes) (SMQ)	20	0.0 (0.0, 1.0)	17.46	1.69–180.07	0.34	0.20–0.56	Early failure

Note: With some cases, one may experience more than one SMQ.

TTO, Time-to-onset; AE, adverse events; TXA, Tranexamic acid; SMQ, Standardized MedDRA Queries; FAERS, The U.S. Food and Drug Administration (FDA)’s Adverse Event Reporting System; IQR, interquartile range; CI, confidence interval.

Among 367 cases with gender data, AE reports were more frequent in females (n = 260) than in males (n = 107) (Fig 4C). Notably, the cumulative incidence of AEs showed a significant difference between males and females. The median onset time for females was 1 day (IQR: 0.0–6.0 days), compared to 0 days (IQR: 0.0–2.0 days) for males (Wilcoxon test, *P* = 0.0014) (Fig 4D).

## Discussion

This study represents the first pharmacovigilance investigation to jointly utilize the FAERS and VigiAccess databases for analyzing TXA-related AEs. To our knowledge, this is among the largest pharmacovigilance analyses of TXA to date, encompassing 17,787 AE reports from two independent databases. By leveraging multidimensional pharmacovigilance tools, we identified demographic trends, AE distributions, and safety signals. Our findings offer a detailed perspective on TXA-related AEs, emphasizing safety considerations for clinical decision-making.

A notable finding was the predominance of AE reports among females, suggesting potential gender differences in AE reporting patterns. TXA is widely prescribed for bleeding-related disorders that disproportionately affect women, such as heavy menstrual bleeding, postpartum hemorrhage, and other gynecological conditions [24]. This widespread use in female-specific conditions may partly explain the observed sex differences. Regarding age distribution, older adults (≥ 65 years) were more represented in FAERS, while younger adults (18–44 years) predominated in VigiAccess. Additionally, differences in reporting culture, indication mix, and the inclusion of non-prescription use across regions may further contribute to this discrepancy. Given these structural differences, findings from the two databases should be interpreted independently, with VigiAccess results serving as exploratory and complementary evidence. The 60% concordance rate between the two databases for PT-level signals suggests reasonable reproducibility of key safety signals across independent pharmacovigilance systems. However, both databases share the inherent limitations of spontaneous reporting, including indication bias and absence of exposure denominators.

Our analysis also revealed a clear temporal trend, with over 50% of AE reports occurring between 2019 and 2024. This aligns with the increasing acceptance and expanded indications for TXA, which has demonstrated well-documented benefits in a variety of clinical settings, not only for trauma, but can also be utilized across multiple specialties to manage hemorrhage [25]. However, the increase of TXA-related AE reports may partly reflect expanded use across broader clinical contexts, which warrants pharmacovigilance attention [26].

The SOC distribution for TXA revealed both similarities and differences between the two databases. In VigiAccess, gastrointestinal disorders, skin and subcutaneous tissue disorders, and general disorders and administration site conditions were the most frequently reported, whereas in FAERS, stronger signals were observed for nervous system disorders and injury, poisoning, and procedural complications. Importantly, some findings were consistent with TXA’s mechanism of action, particularly its role in modulating fibrinolysis [27]. Theoretically, TXA might be expected to increase the risk of thrombosis and subsequent vascular and cardiovascular complications due to its antifibrinolytic activity [28]. However, data from other studies [29–31] suggested no significant association between TXA administration and the rate of thrombosis-related complications, and the protective effects of TXA were maintained. The SOC-level analysis highlights the importance of vigilance in monitoring AEs across multiple organ systems, particularly in high-risk patients with conditions such as active thromboembolic disease or imbalances in thrombosis and hemostasis [26].

The PT-level analysis further identified specific safety concerns associated with TXA. Shared PT signals, such as seizure, PE, and anaphylactic reaction, underscore the necessity of monitoring for neurological complications, thromboembolism, and skin symptoms during TXA treatment. TXA-related seizures may be attributed to its role as a competitive antagonist of the gamma-aminobutyric acid receptor type A (GABAA). TXA crosses the blood–brain barrier and may directly excite neurons, thereby lowering the seizure threshold [32]. In in vitro and animal experiment, higher TXA level in the cerebral spinal fluid, correlated with serum concentration, was associated with the incidence of seizures [32,33]. And indeed, some clinical studies indicated that high-dose TXA administration was linked to a higher incidence of seizures [28,34]. Beyond common PTs, certain serious AEs, like myoclonic epilepsy, renal cortical necrosis, and coronary artery thrombosis, exhibited exceptionally high ROR values. However, these signals were based on relatively small case numbers, and high ROR values with low absolute counts are susceptible to statistical instability; they should therefore be interpreted with caution and regarded as signals warranting further investigation rather than confirmed safety concerns.

It should be noted that TXA is predominantly used in high-acuity clinical settings, including trauma, major surgery, and critical illness, where thromboembolic events (e.g., PE, AMI), cardiac arrest, and MODS are complications of the underlying condition rather than necessarily drug-related effects. The observed disproportionality signals for these PTs may therefore be subject to confounding by indication, whereby the severity of the hemorrhagic condition or the surgical context, rather than TXA exposure itself, contributes to AE reporting. In the absence of indication-restricted analyses or an active comparison (e.g., other hemostatic agents), these signals should be interpreted with caution.

Furthermore, TXA is approved across heterogeneous indications, including heavy menstrual bleeding, surgical hemorrhage prophylaxis, and trauma-associated hemorrhage, with signals pooled across these different clinical contexts. As FAERS does not consistently capture structured indication data for all reports, signals across these indications may obscure indication-specific safety profiles. Future pharmacovigilance studies with indication-stratified analyses would provide more refined signal characterization.

Our study evaluated disproportionality signal differences across subgroups stratified by age, gender, and fatal outcomes. Females showed higher reporting disproportionality for headache and PE, while males exhibited stronger signals for hypertension and GTCS. Older individuals (≥ 65 years) demonstrated higher disproportionality signals for GTCS and atrial fibrillation, whereas younger individuals (< 65 years) showed higher reporting proportions for nausea and rash, and more death-related AEs caused by cardiac arrest and MODS. These differences in reporting patterns may result from individual variations, underlying comorbidities, or differential prescribing patterns. Factors like hormone levels, gastrointestinal physiology, and hepatorenal function may influence drug absorption, distribution, metabolism, and excretion, potentially contributing to sex- and age-related differences in AE profiles [35]. Of note, the death versus non-death comparison is particularly susceptible to confounding. Patients with fatal outcomes likely had more severe underlying conditions, more complex surgical or trauma contexts, and greater exposure to concomitant medications, any of which could independently contribute to the observed AEs. The higher disproportionality signals for cardiac arrest, MODS, and PE in the death group therefore cannot be attributed to TXA exposure per se, and should not be interpreted as evidence that TXA directly causes these fatal events.

TXA-related AEs were characterized by early failure patterns, with over four-fifths of AEs reports occurring within the first month of treatment, and gradually decreased with prolonged exposure time. This finding aligns with TXA’s pharmacokinetics, which exerts its effects rapidly after administration [36]. However, in the context of TXA’s use in perioperative and acute hemorrhagic settings, early-onset AEs may also reflect the severity of underlying conditions, surgical interventions, or concomitant therapies rather than direct drug toxicity. The predominance of 0-day TTO records, while clinically plausible for intravenous administration, may additionally reflect date imprecision or same-day reporting conventions. Therefore, the early failure pattern observed here should be interpreted as a reporting pattern rather than a pharmacological characteristic of TXA. Besides, gender differences were also observed in TTO analysis. Compared with males, females exhibited a longer median TTO, which may reflect sex-based differences in TXA pharmacodynamics or underlying pathophysiology.

The findings of this study have important clinical implications. The disproportionality signals for thromboembolic and neurological events suggest that enhanced monitoring may be warranted, particularly in patients with a history of thrombotic events or seizure disorders. Hypersensitivity reaction signals, including anaphylaxis, suggest the importance of emergency preparedness during TXA administration, particularly in acute settings. Moreover, close monitoring during the initial treatment period may be necessary, as TTO analysis identified a higher AE reports in this period; however, this reflects reporting patterns rather than confirmed incidence rates. Special attention should be focused to high-risk populations, including older adults, females, and individuals with pre-existing comorbidities, to minimize the risk of adverse outcomes.

## Limitations

This study has several limitations. First, the reliance on spontaneous reporting databases introduces potential reporting biases, including underreporting, over-reporting, and incomplete data. Second, substantial missingness was present in key covariates, including route of administration, TTO, and body weight. Analyses involving these variables may introduce selection bias due to limited representativeness of available subsets. In particular, age data were missing for 438 of 1,780 FAERS reports (24.61%), which may have introduced selection bias into age-based subgroup analyses. Third, the median TTO of 0 days may reflect date imprecision, or reporting conventions, particularly given that route of administration was unspecified in nearly half of reports. The early failure pattern should therefore be considered hypothesis-generating. On the other hand, same-day AEs in perioperative settings may reflect the underlying condition or surgical context rather than direct drug effects.

Fourth, VigiAccess provides only aggregated drug–event frequency data without access to individual ICSRs; therefore, VigiAccess-derived signals were based on approximate contingency tables and should be regarded as exploratory findings requiring further validation. Fifth, confounding by indication is a major limitation of this analysis. Many identified signals, including PE, AMI, cardiac arrest, and MODS, are well-recognized complications of trauma, surgery, and critical illness. It is not possible to determine whether these signals reflect true drug effects or are attributable to the severity of the underlying condition. Sixth, signals were pooled across heterogeneous TXA indications (e.g., heavy menstrual bleeding, surgical prophylaxis, trauma), which may mask indication-specific safety patterns.

Seventh, the restriction of tabulated PT-level results to those with ≥10 reports, while consistent with standard practice for statistical stability, may have excluded rarer signals with 3–9 reports that could be clinically meaningful. Future studies with larger datasets may be better powered to characterize low-frequency signals. Additionally, subgroup comparisons involved simultaneous testing across multiple PTs. Although FDR correction was applied, the exploratory nature of these analyses and small subgroup samples limit the reliability of individual PT-level findings. Finally, disproportionality analysis, while valuable for signal detection, cannot provide definitive evidence of a causal (or inverse) relationship between drug exposure and AEs. Further validation from independent data sources, along with insight into potential mechanisms and prevention of TXA-related AEs, are essential to confirm the causal nature of these signals.

## Conclusion

This study provides a comprehensive assessment of TXA-related safety profile based on real-world pharmacovigilance data. By analyzing key demographic trends, SOC and PT distributions, specific high-risk populations, and TTO patterns, we characterized the multidimensionality of TXA-related AE reporting patterns. Thromboembolic, neurological, and hypersensitivity events demonstrated stronger disproportionality signals, emphasizing the importance of close monitoring during the initial period. Subgroup analyses identified differential reporting patterns by age and gender. However, these represent hypothesis-generating signals rather than confirmed differential incidence risks, and should be interpreted in the context of the inherent limitations of spontaneous reporting data. Future studies are warranted to validate these findings and further optimize its clinical application.

## Supporting information

S1 Table Four table of measure of disproportionality. (DOCX)

S2 Table ROR, PRR, MHRA, BCPNN, and MGPS methods, formulas, and thresholds. (DOCX)

S3 Table The READUS-PV checklist. (DOCX)

S1 Fig Annual distribution of AE reports for TXA in VigiAccess. (TIF)

S2 Fig Annual distribution of AE reports for TXA in FAERS. (TIF)

S3 Fig The top 50 Signal strength of AEs at the PT level ranked by ROR for TXA in VigiAccess. (TIF)

S4 Fig The top 50 Signal strength of AEs at the PT level ranked by ROR for TXA in FAERS. (TIF)
