# Supplementary material for: Real‑world safety evaluation of tranexamic acid: Signal detection from FAERS and VigiAccess databases
Source: PLoS One. 2026 Jul 10;21(7):e0353459. doi: 10.1371/journal.pone.0353459 (PMC13353941; doi:10.1371/journal.pone.0353459)
Supplement: S1 Table — (DOCX) [file pone.0353459.s001.docx]

**S1 Table.** Four table of measure of disproportionality.

|  | **Target AEs** | **Non-Target AEs** | **Total** |
| --- | --- | --- | --- |
| TXA | a | b | a+b |
| Non-TXA | c | d | c+d |
| Total | a+c | b+d | n=a+b+c+d |

a = The number of reports of TXA with the adverse event of interest. b = The number of reports of all other drugs with the adverse event of interest. c = The number of reports of TXA with all other adverse events. d = The number of reports of all other drugs with all other adverse events.

Abbreviations: AE, adverse event; TXA, tranexamic acid.
