# Supplementary material for: Real‑world safety evaluation of tranexamic acid: Signal detection from FAERS and VigiAccess databases
Source: PLoS One. 2026 Jul 10;21(7):e0353459. doi: 10.1371/journal.pone.0353459 (PMC13353941; doi:10.1371/journal.pone.0353459)
Supplement: S2 Table — (DOCX) [file pone.0353459.s002.docx]

**S2 Table.** ROR, PRR, MHRA, BCPNN, and MGPS methods, formulas, and thresholds.

| **Method** | **Formula** | **Threshold** |
| --- | --- | --- |
| ROR | $ROR=\frac{\text{（}a/c\text{）}}{(b/d)}=\frac{ad}{bc}$ $SE\text{（}1\text{n}ROR\text{）}=\sqrt{(\frac{1}{a}+\frac{1}{b}+\frac{1}{c}+\frac{1}{\text{d}})}$    95%CI=*e*^1n(ROR)±1.96SE^ | a ≥ 3  95%CI (lower limit) > 1 |
| PRR | $PRR=\frac{\text{a}/\text{（}a+b\text{）}}{c/(c+d)}$ $SE\text{（}1\text{n}PRR\text{）}=\sqrt{(\frac{1}{\text{a}}-\frac{1}{a+b}+\frac{1}{c}-\frac{1}{c+d})}$    95%CI=*e*^1n(PRR)±SE^ | a ≥ 3  95%CI (lower limit) > 1 |
| MHRA | $\chi2 =\frac{{(ad-bc)}^{2}(a+b+c+d)}{( a+b)(a+c)(c+d)(b+d)}$ | a ≥ 3  $\chi2\geq4$ |
| BCPNN | $IC=\log_{2}\frac{p(x,y)}{p(x)p(y)}$=  E(IC)=${log}_{2}\frac{(a+\gamma11)(a+b+c+d+\alpha)(a+b+c+d+\beta)}{（a+b+c+d+\gamma）(a+b+\alpha1)(a+c+\beta1)}$  V(IC)=  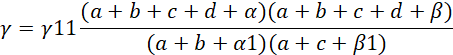    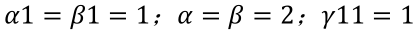 | IC025>0 |
| MGPS | 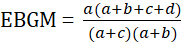  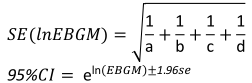 | EBGM05>2 |

Abbreviations: ROR, reporting odds ratio; PRR, proportional reporting ratio; MHRA, Medicines and Healthcare products Regulatory Agency; BCPNN, bayesian confidence propagation neural network; MGPS, multi-item gamma Poisson shrinker; EBGM, empirical Bayesian geometric mean; CI, confidence interval; χ2, chi-squared; IC, information component; IC025, the lower limit of the 95% one-sided CI, of the IC; EBGM05, the lower 95% one-sided CI, of EBGM.

Equation: a = The number of reports of tranexamic acid with the adverse event of interest. b = The number of reports of all other drugs with the adverse event of interest. c = The number of reports of tranexamic acid with all other adverse events. d = The number of reports of all other drugs with all other adverse events.
