# Supplementary figures and images for: Real‑world safety evaluation of tranexamic acid: Signal detection from FAERS and VigiAccess databases
Source: PLoS One. 2026 Jul 10;21(7):e0353459. doi: 10.1371/journal.pone.0353459 (PMC13353941; doi:10.1371/journal.pone.0353459)

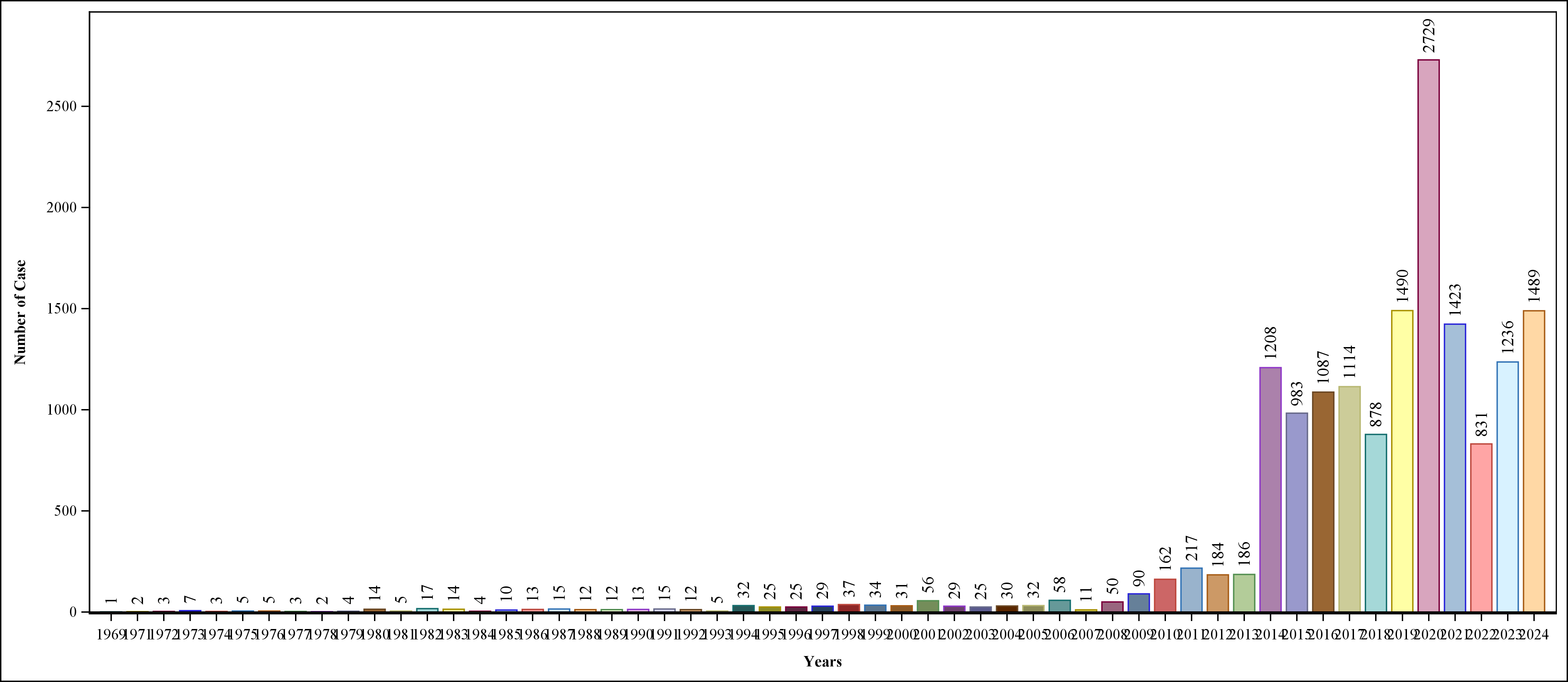

Supplement: S1 Fig — (TIF) [file pone.0353459.s004.tif]

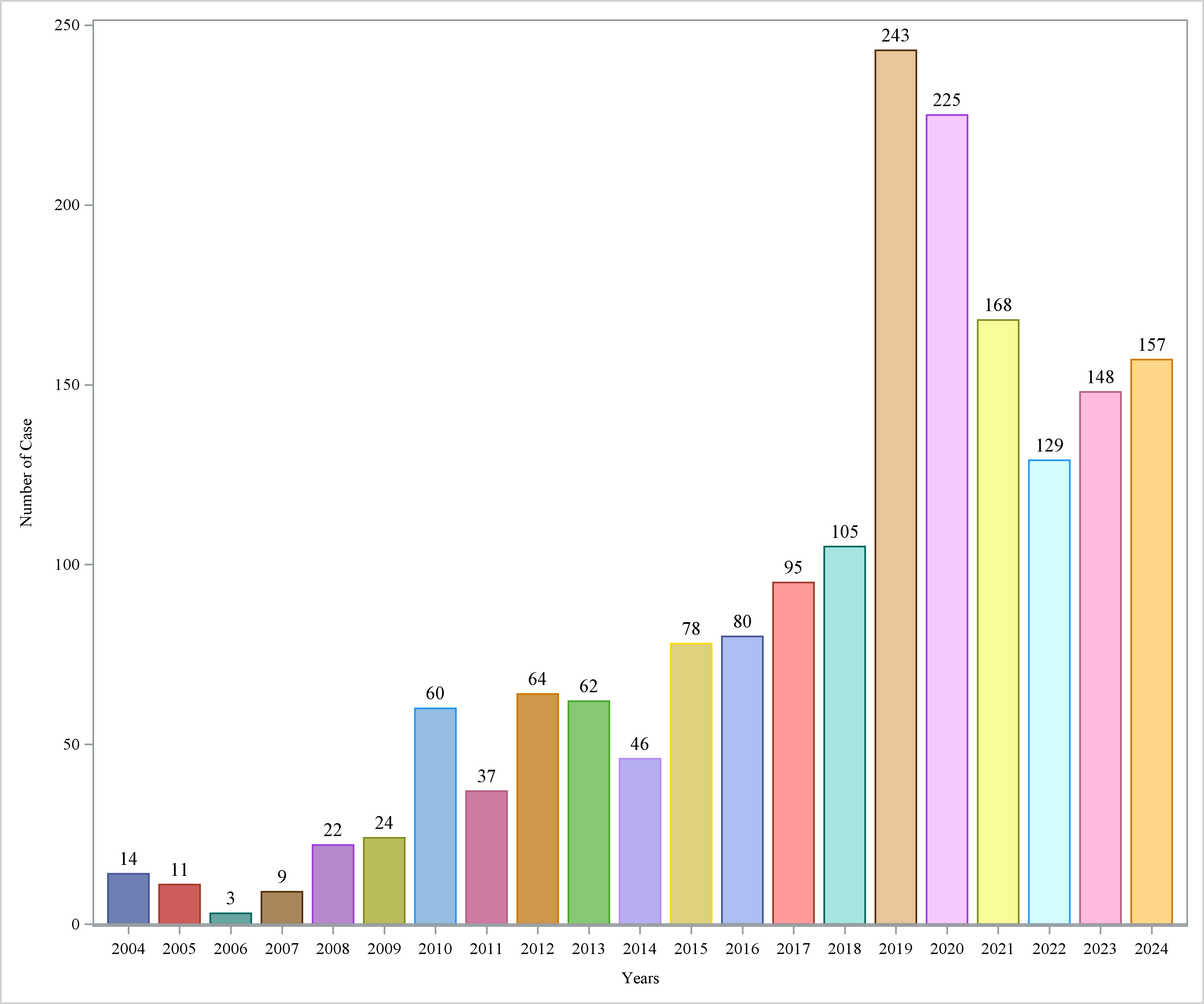

Supplement: S2 Fig — (TIF) [file pone.0353459.s005.tif]

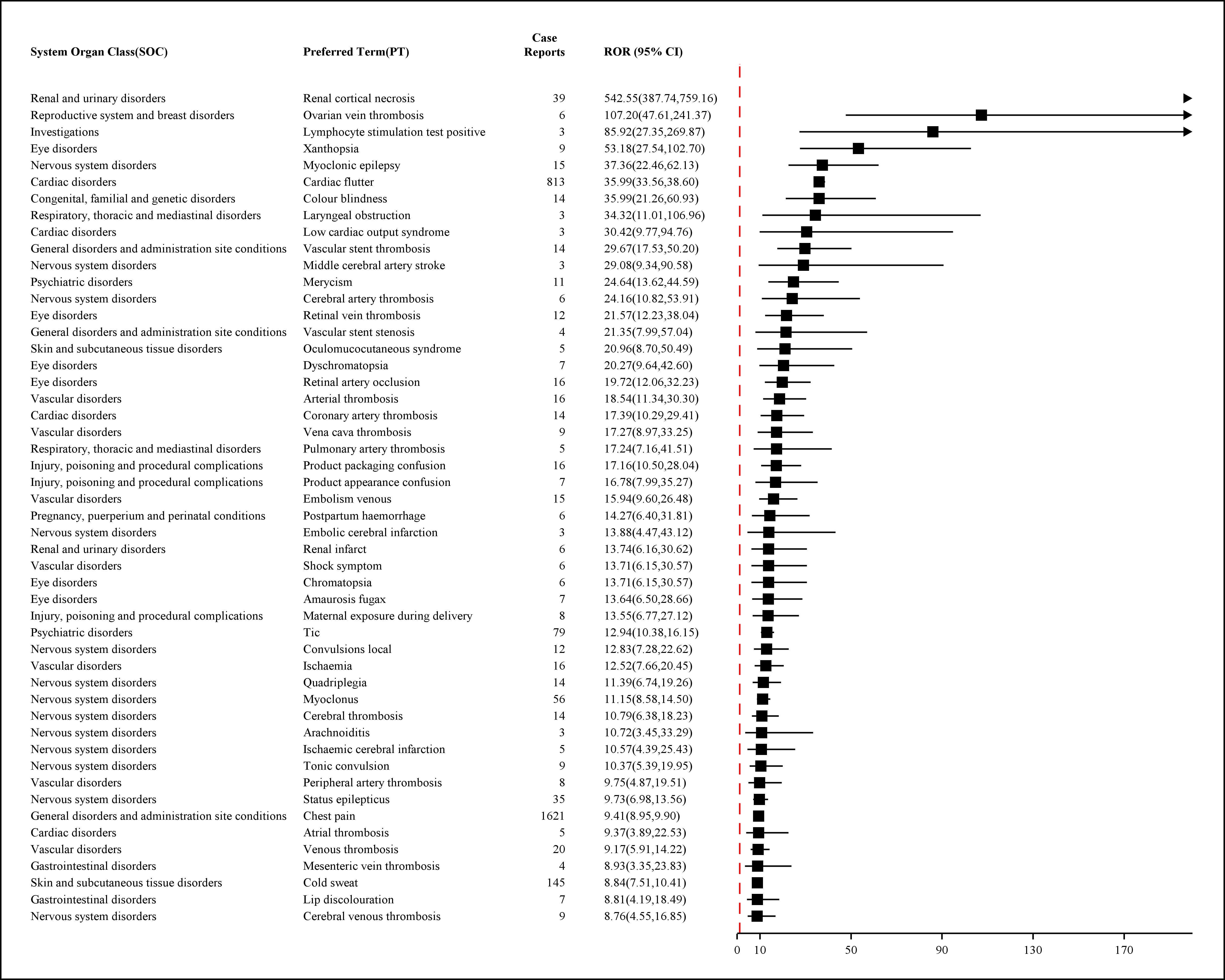

Supplement: S3 Fig — (TIF) [file pone.0353459.s006.tif]

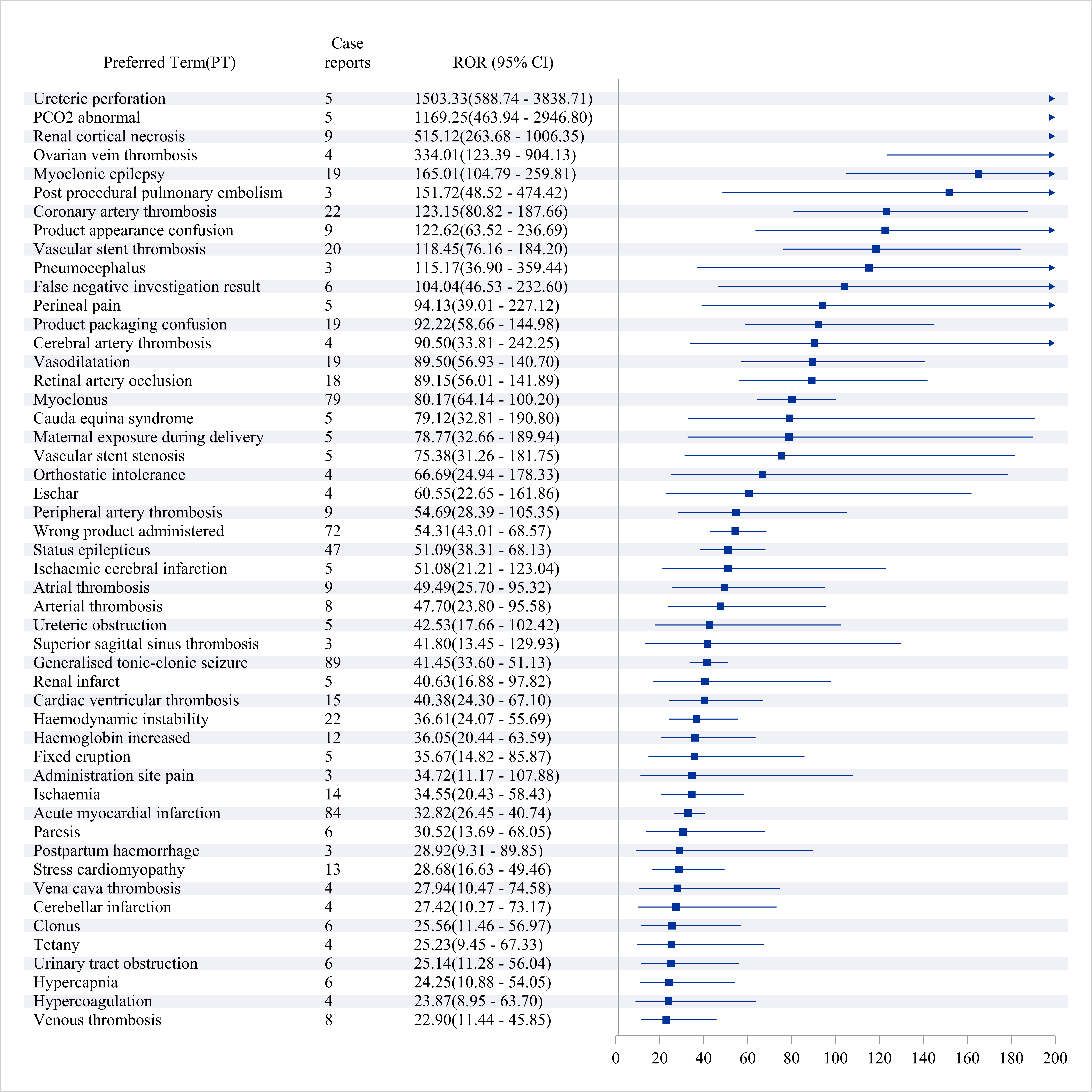

Supplement: S4 Fig — (TIF) [file pone.0353459.s007.tif]
